# Case Report: A Previously Healthy Young Woman With Lethal, Unremitting Metabolic Acidosis: Could a Novel Variant in *ALAS2* Be the Culprit?

**DOI:** 10.1155/crip/1311428

**Published:** 2026-08-31

**Authors:** Greg Brown, Stephen Brown

**Affiliations:** ^1^ Laboratory, Daviess Community Hospital, Washington, Indiana, USA; ^2^ Department of Obstetrics, Gynecology and Reproductive Sciences, Larner College of Medicine, University of Vermont, Burlington, Vermont, USA, uvm.edu

## Abstract

**Background:**

Heme synthesis is critical for several biological processes, including mitochondrial energy production and oxygen delivery via hemoglobin. The initial and rate‐limiting step in heme synthesis is the conjugation of glycine with succinyl‐CoA to form 5‐aminolevulinic acid (ALA), which is catalyzed by two closely related enzymes that are coded by highly homologous genes, known as *ALAS1* and *ALAS2*. Loss‐of‐function variants in *ALAS2* result in sideroblastic anemia, whereas gain‐of‐function variants result in porphyria. We present a case of a woman who died with severe metabolic acidosis and was found to have a rare variant in *ALAS2*. We propose the hypothesis that the *ALAS2* variant, in conjunction with other factors, was responsible for her demise.

**Case Report:**

A previously healthy 34‐year‐old woman presented to the emergency department of her local hospital complaining of severe fatigue. Her arterial pH was 6.95. No cause for her acidosis could be identified, and she expired from unremitting acidosis. The autopsy was notable for an enlarged liver. Whole exome sequencing identified a rare, paternally inherited variant in *ALAS2*. This prompted a re‐evaluation of the autopsy and the development of a novel hypothesis to explain her acidosis. We propose that the *ALAS2* variant identified in this family, NM_000032.5(*ALAS2*)c.1273C>T(p.Pro425Ser), is a conditional allele that can contribute to mitochondrial dysfunction under unusual conditions.

**Discussion:**

We hypothesize that the P425S variant identified in *ALAS2* leads to dysfunction of the homeostatic down‐regulation of *ALAS2* in times of cofactor deficiency. This case illustrates that DNA sequencing as a part of the autopsy procedure will, as it becomes more routine, lead to the identification of sequence variants that may or may not be clinically relevant. The pursuit of hypotheses, prompted by genetic data, has the potential to identify novel pathogenic mechanisms.

## 1. Introduction

Metabolic acidosis can arise from many different mechanisms, both inherited and acquired. Unexplained acidosis in a healthy adult is rare. In such cases, clinicians are quick to rule out toxic exposures, sepsis, and routine metabolic issues such as diabetic ketoacidosis [1]. Inborn errors of metabolism, while often a cause of acidosis in babies, would be extremely rare in adults [2, 3]. In this report, we present a previously healthy woman in her mid‐30s with new onset of severe metabolic acidosis. No underlying cause for her acidosis was identified, and despite heroic efforts to correct her metabolic abnormalities, she developed multiple organ failure and died. Because of the many questions surrounding her clinical course, postmortem studies included whole‐exome sequencing of the woman and her parents. This revealed a paternally inherited, extremely rare missense variant in *ALAS2*. Prompted by the *ALAS2* variant, as well as the highly unusual premortem finding of ring sideroblasts on her peripheral blood smear, we reviewed the autopsy findings and obtained iron stains on various tissues. The combination of histopathologic findings is striking and leads us to propose the hypothesis that this patient′s demise was due to a unique combination of factors, including anemia, pyridoxine deficiency, and the presence of the *ALAS2* variant.

*ALAS1* and *ALAS2* are highly homologous genes that code for aminolevulinic acid synthase 1 and 2, which catalyze the initial, rate‐limiting step in heme synthesis [4–6]. *ALAS1* is ubiquitously expressed and provides heme for electron transport, whereas *ALAS2* is expressed only in the erythrocyte lineage and provides heme for hemoglobin. The demand for erythrocyte heme is at least an order of magnitude greater than the demand for heme in energy metabolism. Loss‐of‐function variants in *ALAS2* (which is X‐linked) result in sideroblastic anemia (OMIM 300751), whereas gain‐of‐function variants can result in porphyria (OMIM 300752). One‐third of X‐linked sideroblastic anemia patients are females, which is attributed to skewed X‐inactivation [7]. Females tend to present later, in the third or fourth decade, and have a milder illness [8]. *ALAS1* variants are not associated with any known human illnesses.

In this report, we propose the hypothesis that the *ALAS2* variant identified in this patient does not result in loss‐of‐function or gain‐in‐function in any simple way. Rather, we posit that it is a conditional allele that becomes critically important in the setting of severe pyridoxine deficiency, which, theoretically, could result from blood loss and persistent, inappropriate hematopoiesis despite worsening deficiency. Although there is no precedent for this idea in the human literature, a recent publication describes a pyridoxine‐dependent variant in mouse *Alas2* that results in lethality [9].

## 2. Case Report

**Figure 1:**
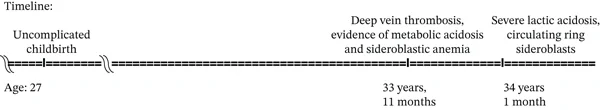
Timeline depiction of the patient′s medical history.

An otherwise healthy 34‐year‐old White woman reported poor stamina for at least a year prior to presentation. Notably, she had a completely normal pregnancy and delivery 7 years earlier, attesting to her prior good health. Two months before admission, she was diagnosed with deep vein thrombosis and treated with apixaban. At that time, she reported severe, debilitating fatigue, hypersomnia, mood swings, and anxiety. She was not taking any other medications and denied the use of any illicit drugs or excessive alcohol intake. Initial laboratory values revealed a macrocytic anemia with a hemoglobin of 9.7 g/dL (12.0–16.0), MCV 115 fL (80–94), striking anisopoikilocytosis with RDW‐SD 91.4 fL (36–55), and probable metabolic acidosis with an anion gap of 23 mmol/L (5–15). Liver enzymes were elevated (AST 518 U/L, ALT 137 U/L, and alkaline phosphatase 209 U/L), consistent with hepatobiliary inflammation. Nucleated red blood cells were determined to be 0.5 per 100 WBCs by automated count. See Figure 1 for a timeline depiction of the patient′s medical history.

**Figure 2:**
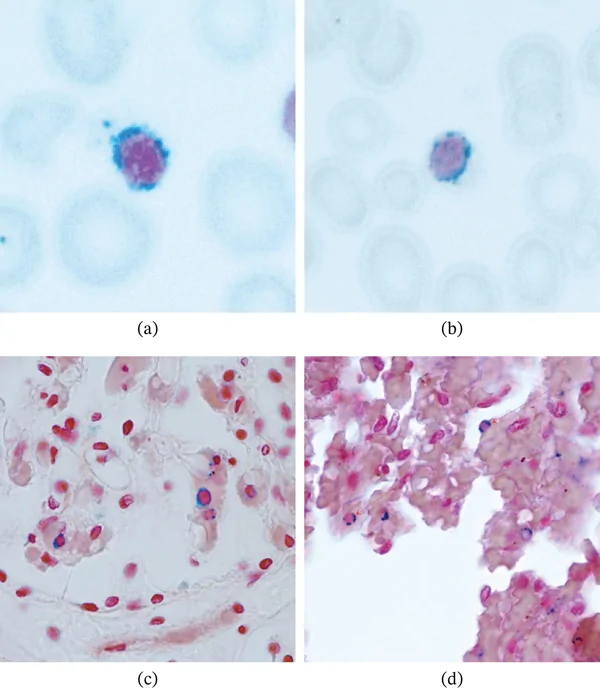
(a,b) Peripheral blood smears showing nucleated erythroid precursors with perinuclear iron granules, confirming ring sideroblast morphology (Prussian blue, ×1000). (c) Renal vessels with ring sideroblasts (Prussian blue, ×400). (d) Spleen with ring sideroblasts (Prussian blue, ×400).

Upon presentation to the emergency department of her local hospital 2 months later, she was profoundly acidotic (arterial pH 6.935) with a venous lactate of 34 mmol/L, and was admitted to the ICU. A venous blood gas revealed a pO_2_ of 80.5 mmHg (35.0–45.0), indicating marked impairment of mitochondrial oxygen consumption. The peripheral smear showed numerous Pappenheimer bodies, nucleated erythrocytes (54.9 per 100 WBCs by automated count), and cells with perinuclear iron granules. Subsequently, Prussian blue staining confirmed circulating ring sideroblasts (Figure 2a,b). This highly unusual finding was reported by the hematology laboratory but was not pursued further. Extensive evaluation for acquired causes of lactic acidosis (including sepsis, toxic alcohols, cyanide, and carbon monoxide poisoning, as well as endocrine and metabolic disturbances) was unrevealing.

Amino acid levels revealed a homocystine of 4.5 *μ*mol/L (0.0–0.2 *μ*mol/L)—more than 20 times the stated upper limit. Homocystine is the disulfide dimer of homocysteine and is the form of homocysteine routinely assayed in amino acid panels [9]. Therefore, it is present at a much lower concentration than total homocysteine, which is determined by preanalytical reduction of other disulfides that homocysteine normally forms with various plasma components, principally the sulfur‐containing amino acids of albumin [10]. All causes of elevated homocystine (B12 deficiency, *MTHFR* gene variants, kidney disease, and alcohol or tobacco use) [11] were ruled out, establishing pyridoxine deficiency as the most likely cause [12].

A cyanide level was below the detection limit. Carbon monoxide poisoning was excluded by arterial blood gas testing. Lipase was markedly elevated (up to 5545 U/L), but clinical and radiologic findings were not consistent with pancreatitis. The patient deteriorated despite aggressive care. After 36 h in the community hospital, she was transferred to a tertiary care facility, where she died 36 h later. At no point did her acidosis show more than minimal improvement.

### 2.1. Postmortem Findings

The external exam noted obesity (length = 185 cm, weight = 109.1 kg, and BMI = 32) and scleral icterus. The principal gross finding was hepatic enlargement (two times normal weight). Histologically, there was steatosis without ballooning or other evidence of steatohepatitis. Along with diffuse fibrin thrombi, necrosis and infarcts were observed in all organs (including the pancreas), which were attributed to disseminated intravascular coagulation.

Several months after completion of the autopsy report, the results of whole‐exome sequencing were reported. This identified heterozygosity for an extremely rare variant in NM_000032.5(*ALAS2*):c.1273C>T, p.(P425S). This single‐base change, which was inherited from the patient′s father, changes the proline at Position 425 to a serine, and was classified by the testing lab, GeneDx, as a variant of uncertain significance, based on the following: (i) it had not been previously published as either benign or pathogenic; (ii) that it was extremely rare; and (iii) that software tools predicted it to be deleterious. At the time of the exome report, this variant did not appear in ClinVar. At the time this manuscript was prepared, there was a single entry in ClinVar (ClinVar Variation ID: 3253016), which represents this patient. Version 4.1.1 of gnomAD reports four instances of this variant out of a total of 1,207,135 sequences, corresponding to an allele frequency of 3.32e‐6 (4/1,207,135 = 0.00000332 = 3.32e − 6). Of the four instances, one was present in an elderly male. The REVEL score [13] of 0.954 associated with this variant supports, but cannot prove pathogenicity [17].

The finding of the *ALAS2* variant, taken together with the previously noted finding of ring sideroblasts in the peripheral smear, prompted a reconsideration of the autopsy, especially the histopathology.

**Figure 3:**
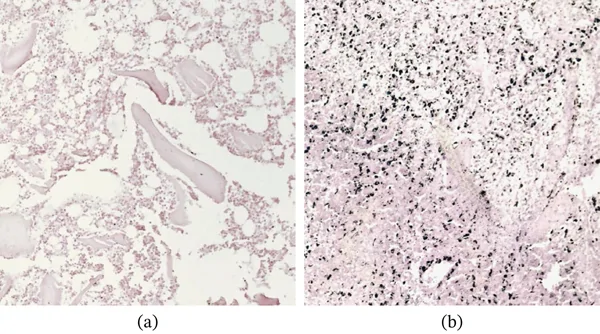
(a) Bone marrow showing absence of storage iron (Prussian blue, ×400). (b) Spleen with siderosis (Prussian blue, ×400).

**Figure 4:**
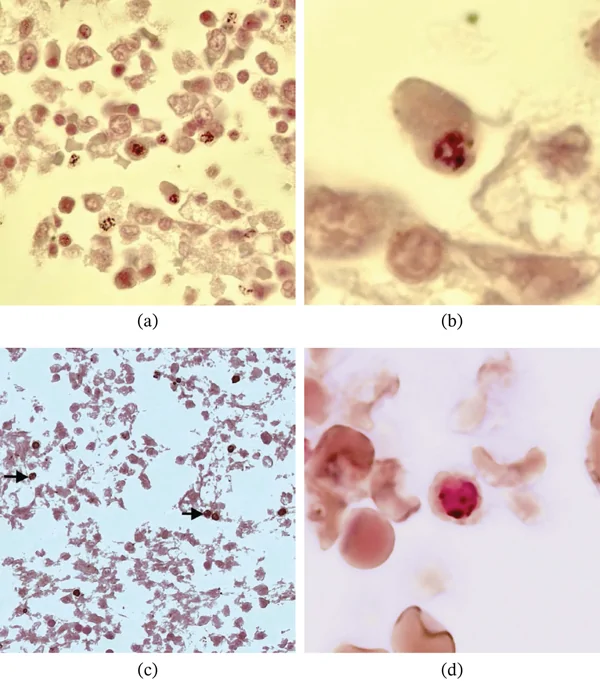
(a) Bone marrow showing scattered cells with black staining in nuclei (Prussian blue, ×400). (b) Higher magnification of cell slightly below the middle in Panel a (Prussian blue, ×1000). (c) Spleen with black staining in nuclei of scattered cells (Prussian blue, ×400). (d) Intravascular erythroblast with black staining in nucleus (Prussian blue, ×1000).

Review of routine histopathology was notable for complete absence of iron within the bone marrow. In contrast, there was siderosis of the spleen (Figure 3a,b). Figure 4a,b show that with Prussian blue stain, there is black staining in nuclei of erythroid precursors within the marrow. Perinuclear blue staining of ring sideroblasts could not be identified in the marrow, despite exhaustive searching. Figure 4c shows extensive black staining of nuclei in the spleen. Similar black nuclear staining is rarely identifiable in intravascular nucleated red blood cells (Figure 4d). (See Section 3 for discussion of the significance of the black staining).

**Figure 5:**
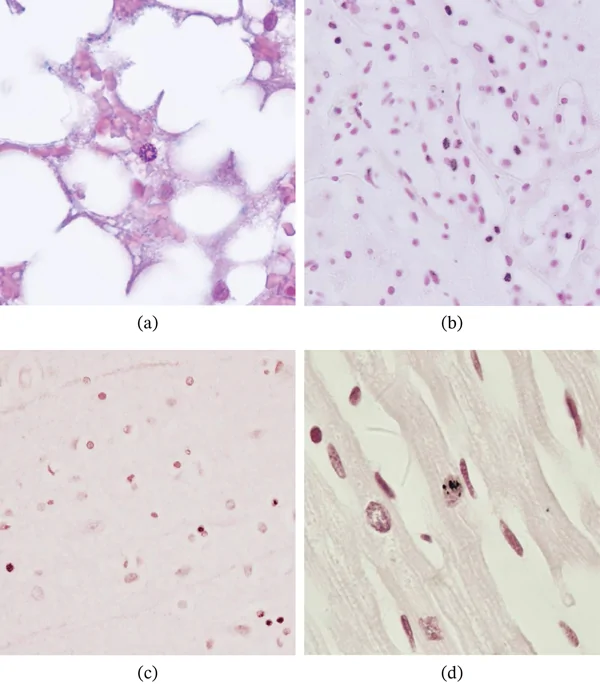
(a) Hepatocyte with black staining of nucleus (Prussian blue, ×500). (b) Renal tubular cells with black staining of nuclei (Prussian blue, ×400). (c) Cerebral cortical cells with black staining of nuclei (Prussian blue, ×400). (d) Myocardial cell with black staining of nucleus (Prussian blue, ×500).

**Figure 6:**
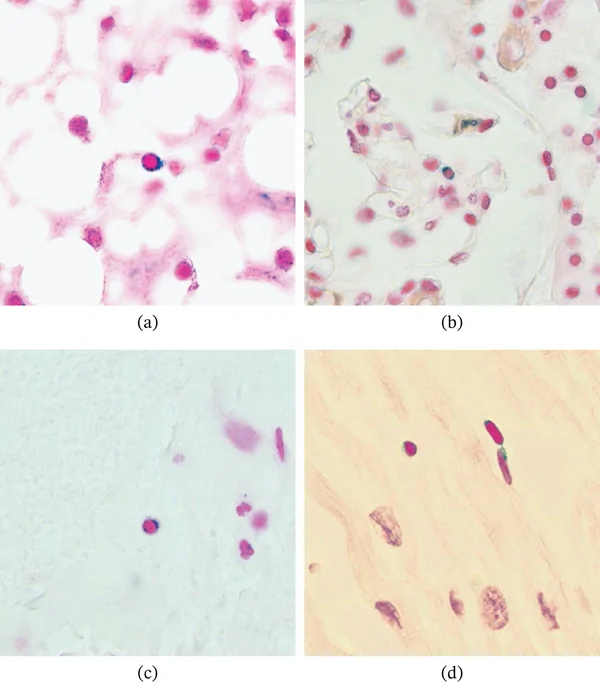
(a) Hepatocyte with blue granules around nucleus (Prussian blue, ×400). (b) Renal tubular cell with blue granules around nucleus (Prussian blue, ×400). (c) Cerebral cortical cells with blue granules around nuclei (Prussian blue, ×400). (d) Myocardial cells with blue granules around nuclei (Prussian blue, ×500).

**Figure 7:**
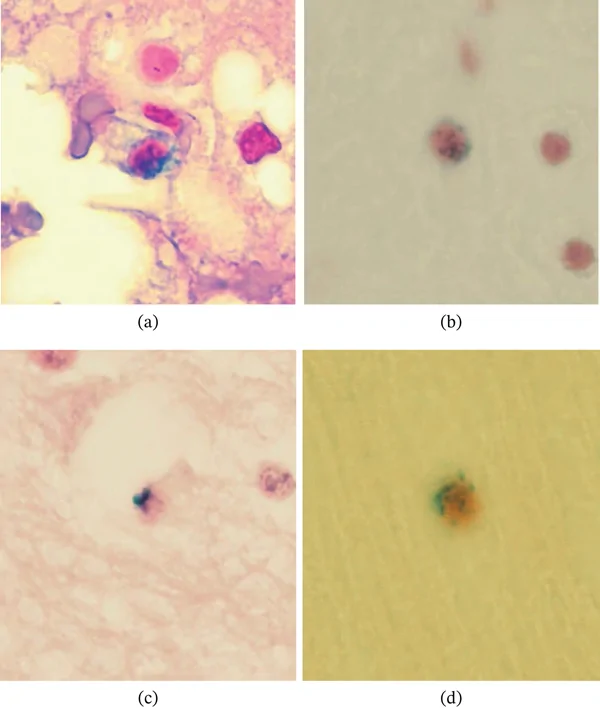
(a) Hepatocyte with both black staining in and blue granules around nucleus (Prussian blue, ×1000). (b) Renal tubular cell with both black staining in, and blue granules around nucleus (Prussian blue, ×1000). (c) Cerebral cortical cell with both black staining in, and blue granules around nucleus (Prussian blue, ×400). (d) Myocardial cell with both black staining in, and blue granules around nucleus (Prussian blue, ×1000).

In Figure 5, iron stains show black nuclear staining—identical to that in erythroid cells—in parenchymal cells of the liver, kidney, brain, and heart. In Figure 6, there is blue perinuclear staining in the same organs, reminiscent of erythroid ring sideroblasts, and in Figure 7, there are cells in the same organs with both black nuclear and blue perinuclear staining.

## 3. Discussion

The goal of postmortem examination is to increase knowledge and understanding of disease mechanisms. In this case, the officially reported autopsy did little to shed light on why this young woman died. In this case report, we present a novel hypothesis that makes sense out of the highly unusual histopathologic and laboratory findings, and, more importantly, may also explain why this patient died.

The clinical history indicates that this young woman was completely healthy until about 1 year prior to her death. She reported the insidious onset of fatigue, and, in retrospect, was suffering from chronic metabolic acidosis, as evidenced by her elevated anion gap 2 months prior to her final presentation (See Section 2 above for numerical values). At that time, she had significant macrocytic anemia, anisocytosis with elevated RDW possibly suggesting sideroblastic anemia, and rare circulating nucleated RBCs. Although premenopausal women are frequently anemic, the striking red cell parameters and presence of nucleated RBCs in this patient pointed to an unusual process. Two months later, nucleated red blood cells were markedly elevated, and there were ring sideroblasts in her peripheral smear, a finding which, as far as we are aware, has never been previously reported.

The clinicians who cared for this patient were preoccupied with her profound metabolic abnormalities and did not pursue the hematologic findings. In retrospect, the most common causes of acquired sideroblastic anemia [14] (heavy alcohol use, isoniazid, and other medications) were excluded by history and there was no evidence in peripheral blood or postmortem marrow of myelodysplasia. She had no vocational or avocational activity that would have led to metal toxicity, no history of bowel surgery or other cause of malabsorption, and no significant exposure to unregulated water (such as from a private well). However, metal (lead, zinc, and arsenic) toxicity and copper deficiency were not formally excluded as possible causes of sideroblastic anemia. Her ceruloplasmin was 20 mg/dL (20–60 mg/dL).

In our re‐evaluation of the autopsy, iron staining of multiple tissues revealed several significant findings. Considering that ring sideroblasts normally are confined to the bone marrow, and this patient had them in her circulating blood, we expected to find ring sideroblasts in her marrow as well. However, none could be found despite repeated meticulous searches. Although storage iron was absent in the marrow, positive (black) iron staining could be identified within nuclei in scattered cells (Figure 4), most of which were clearly erythroid precursors (Figure 4b). Further, in tissue sections, the presence of ring sideroblasts within vessels in Figure 2c, and in the spleen in Figure 2d, was consistent with their presence in the premortem peripheral blood smear (Figures 1b and 2a).

Unequivocal black nuclear staining could also rarely be identified in intravascular erythroblasts (Figure 4d), and frequently in the spleen (Figure 4c). We believe that there is a continuum of both placement and morphology, beginning with indisputable premortem peripheral smear ring sideroblasts, to marrow erythroid cells with black nuclear granules to the presence of both morphologies in vessels and spleen. To support this claim, we examined ring sideroblasts in archived cases of sideroblastic anemia and found black staining material in the nuclei of almost all sideroblasts. We doubt that our observations are anomalous. The presence of black nuclear material is frequently subtle. Others may have either ignored it or considered it unworthy of mention. We conclude that the black nuclear staining in our case represents an exaggerated form of a typical feature that has been ignored in ring sideroblasts.

We note that cells with identical black nuclear material were identified in multiple tissues, including the liver, kidney, brain, and heart (Figure 5). In the same organs, a few cells with blue deposits could be identified outside the nucleus (Figure 6), and rare, presumably transitional forms with both black nuclear and blue perinuclear deposits were also present (Figure 7).

Another relevant finding was absent marrow iron stores, whereas there was a striking abundance of iron in the spleen. We believe this was a consequence of ineffective erythropoiesis, which was evidenced by the presence of 54.9 nucleated RBCs per 100 WBC. Together with the undetectable haptoglobin, these findings suggest unopposed erythropoietin stimulation with maximal erythropoiesis, premature release of erythroid precursors, and their immediate culling by the spleen.

As noted above, exome sequencing was performed on the deceased patient, and her parents revealed a paternally inherited sequence variant in *ALAS2* that is predicted to result in the highly conserved proline normally present at Position 425 to be converted to serine (P425S), a change that is strongly predicted to alter protein function [13]. Despite such predictions, this patient and her family members provide strong evidence that the P425S variant does not confer any obvious loss of function, since neither the patient′s father nor her son (who inherited the variant) have any evidence of SA. Approximately 1 year after the patient′s demise, the father sought medical attention. At the age of 61, his complete blood count was normal, with hemoglobin 14.2 (13.4–17.1), MCV 84.7 (80.0–94.0), RDW‐SD 42.6 (36.0–55.0), and nucleated RBC 0.00 (0.00–0.00).

Interestingly, the patient′s sister (who also carries the P425S variant of *ALAS2*) recently had an episode of GI bleeding, which apparently precipitated a response not unlike her deceased sister′s, although less severe. After 2 months of pyridoxine supplementation, Pappenheimer bodies (iron granules) and nucleated RBCs were undetectable. (See Table 1).

**Table 1:** Comparison of selected blood counts and chemistries for (in left column), the patient′s sister following episode of bleeding; (middle column), patient′s sister after pyridoxine supplementation; and (right column) the patient two months before death.

	Patient′s sister, after GI bleed	Patient′s sister, after pyridoxine	Patient 2 months before death
Hemoglobin (g/dL)	8.4 (12–16)	9.6	9.7 (12–16)
MCV (fL)	98.6 (80–94)	87.2	115 (80–94)
RDW (fL)	73 (33–55)	49.9	91.4 (36–55)
Nucleated RBCs (%)	0.2 (0.0–0.0)	0.0	0.5 (0.0–0.0)
Anion gap (mmol/L)	20 (10–20)	18	23 (5–15)
Lactate (mmol/L)	1.8 (0.5–2.0)	Not determined	Not determined
Pappenheimer bodies	Present	Absent	Present

Sideroblastic anemia is indicative of a deficiency of the mitochondrial heme precursor, protoporphyrin IX, and therefore hemoglobin. Lactic acidosis with elevated venous pO_2_ is indicative of severely compromised mitochondrial oxidative phosphorylation. However, what is lacking is an explanation of why her mitochondrial function deteriorated. In considering how a variant in *ALAS2* could result in mitochondrial dysfunction after 34 years of good health, we noted that *ALAS1* and *ALAS2* catalyze the same initial step in heme synthesis. However, the demand for heme in the production of erythrocytes is at least 10 times greater than the demand for mitochondrial heme for energy production. In times of cofactor (pyridoxine) deficiency, there must be a mechanism that curtails red cell production in order to spare general mitochondrial function. We propose that the P425S disrupts this aspect of *ALAS2* function. In times of sufficient substrate availability, P425S does not seem to have negative consequences. However, in the setting of severe pyridoxine deficiency, we hypothesize that P425S disrupts the normal mechanism that curtails *ALAS2* activity to make sure that the cofactor is available for energy metabolism. In this scenario, *ALAS2* “steals” critical cofactor from *ALAS1*. The resulting failure of mitochondrial heme production precipitates acidosis. Although this is clearly hypothetical, it could be tested in an animal model.

In their mouse model of X‐linked sideroblastic anemia, Ducamp et al. [15] demonstrate lethality conditioned upon pyridoxine restriction, at least in male hemizygotes. Females, with far milder manifestations, were not tested. In a mouse model of sideroblastic anemia due to mutation in the autosomal gene for a mitochondrial glycine transporter, Slc25a38, extreme sensitivity to pyridoxine restriction was attributed to possible compromise of pyridoxine dependent processes unrelated to heme synthesis, such as a defective response to oxidative stress. Implied is the existence of some, likely multiple nonrandom mechanisms for allocation of pyridoxine to its many purposes, surely to include the direct interaction between pyridoxine and *ALAS1* and *ALAS2*.

This simple hypothesis has the advantage of explaining all available clinical, hematologic, chemical, morphologic, and genetic information about this otherwise mysterious patient. Undoubtedly, there are alternative, equally valid explanations.

## Funding

No funding was received for this manuscript.

## Conflicts of Interest

The authors declare no conflicts of interest.

## Data Availability

The data that support the findings of this study are available on request from the corresponding author. Iron stained slides are available upon request.
